# Homogeneity of GAFCHROMIC EBT2 film among different lot numbers

**DOI:** 10.1120/jacmp.v13i4.3763

**Published:** 2012-07-05

**Authors:** Hirokazu Mizuno, Yutaka Takahashi, Atsushi Tanaka, Takamitsu Hirayama, Tsuyoshi Yamaguchi, Hiroaki Katou, Keiko Takahara, Yoshiaki Okamoto, Teruki Teshima

**Affiliations:** ^1^ Department of Radiation Therapy Osaka Police Hospital Tennoji‐ku Osaka City; ^2^ Department of Medical Physics and Engineering Osaka University Graduate School of Medicine SuitaCity Japan; ^3^ Department of Radiation Oncology Osaka University Graduate School of Medicine SuitaCity Japan

**Keywords:** homogeneity, EBT2, lot, gray value, netOD

## Abstract

EBT2 film is widely used for quality assurance in radiation therapy. The purpose of this study was to investigate the homogeneity of EBT2 film among various lots, and the dose dependence of heterogeneity. EBT2 film was positioned in the center of a flatbed scanner and scanned in transmission mode at 75 dpi. Homogeneity was investigated by evaluating gray value and net optical density (netOD) with the red color channel. The dose dependence of heterogeneity in a single sheet from five lots was investigated at 0.5, 2, and 3 Gy. Maximum coefficient of variation as evaluated by netOD in a single film was 3.0% in one lot, but no higher than 0.5% in other lots. Dose dependence of heterogeneity was observed on evaluation by gray value but not on evaluation by netOD. These results suggest that EBT2 should be examined in each lot number before clinical use, and that the dose calibration curve should be constructed using netOD.

PACS number: 87

## I. INTRODUCTION

GAFCHROMIC EBT film (ISP Corporation, Wayne, NJ) has high spatial resolution and can be handled without processing in a darkroom, allowing artifacts associated with the chemical processing of radiographic films to be eliminated.^(^ ^1^ ^)^ Recently, GAFCHROMIC EBT film has been replaced by a newer version, EBT2, which incorporates a yellow marker dye in the active layer to protect this layer from ambient light exposure. EBT2 was designed for intensity‐modulated radiotherapy (IMRT) quality assurance (QA) applications^(^ ^1^ ^–^ ^4^ ^)^ and has been widely used in clinical practice because of its advantage of weak energy dependence with regard to both radiation energy and type including photon, electron, and proton beams.^(^ ^5^ ^,^ ^6^ ^)^ However, many studies have demonstrated the need for particular attention in the use of EBT2 film due to uncertainties regarding the influence of scanning orientation, nonuniformity of scanners, film development time, and film uniformity.^(^ ^7^ ^,^ ^8^ ^)^ In particular, several papers have demonstrated significant effects on film uniformity in earlier batches of this film.^(^ ^7^ ^,^ ^9^ ^)^ Among these, Hartmann et al.^(^ ^9^ ^)^ reported that EBT2 film had local heterogeneity with dose differences of up to ±6% at 1 Gy, while Aland et al.^(^ ^7^ ^)^ reported uncertainty of 3.8% arising from film heterogeneity in an earlier batch of EBT2 film. Although planar dose distributions are often assessed using gamma criteria of 3%/3 mm,^(^ ^10^ ^)^ film heterogeneity might require larger gamma criteria. To solve the problem of film heterogeneity, Kairn et al.^(^ ^11^ ^)^ demonstrated that EBT2 film should be scanned before and after irradiation without the use of blue channel correction to reduce the effect of local heterogeneity on dose measurements. To our knowledge, however, neither variation in local heterogeneity among several lot numbers, nor the effect of the dose dependence of film heterogeneity, has been reported.

In this study, we investigated film heterogeneity in five lots of GAFCHROMIC EBT2 film. We also investigated the dose dependency of heterogeneity.

## II. MATERIALS AND METHODS

### A. GAFCHROMIC EBT2 film preparation

**Figure 1:**
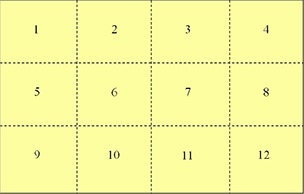
Numbering schema for section of film sheets. The small cut marking is at the upper right side in this schema.

EBT2 film from lots F12170902A, A052810‐02AA, A08161005A, A09171002, and A05131001B was defined as lots A, B, C, D, and E, respectively (Table 1). Three sheets from each lot were used to investigate film homogeneity, and two were used to determine the dose dependency of heterogeneity. The sheets were cut into 12 pieces (6.4×6.8 cm) each and numbered according to the schema shown in Fig. 1.

**Table 1:** Lot number list of EBT2 films used in this study.

	*Lot Number*	*Expiration Date*
Lot A	F12170902A	2011.12
Lot B	A052810‐02AA	2012.5
Lot C	A08161005A	2012.5
Lot D	A09171002	2012.8
Lot E	A05131001B	2012.9

### B. Irradiation

Each piece of film was placed between solid water slabs at a depth of 10 cm and an SSD of 90 cm. Irradiation was performed using a 10× 10cm field with a 10 MV beam with a Varian Clinac 21C/D (Varian, Palo Alto, CA). To investigate intra‐ and intersheet uniformity, three sheets from five lots were exposed to 2 Gy. To investigate the dose dependency of heterogeneity, a single sheet from each of five lots was irradiated at 0.5 Gy and 3 Gy, in addition to 2 Gy. All irradiated films were digitized 24 hr after exposure.^(^ ^12^ ^)^ The irradiated and nonirradiated films were stored at room temperature in a lightproof box.

### C. Film analysis

Digitization was done using an Epson GT‐X970 flatbed scanner (Seiko Epson Corp., Nagano, Japan). Saur and Frengen^(^ ^13^ ^)^ reported that the central area of the scanner bed should be used to achieve the most uniform response. In addition, scanner uniformity has been reported to be dependent on the orientation of the film, due to the different light scatter conditions created by the structure of film's active component.^(^ ^13^ ^)^ To minimize possible confounding by these factors, all pieces were positioned in the center of the scanner bed and scanned in the same side and landscape (original long axis of film parallel to short axis of scan bed) orientation.^(^ ^14^ ^)^ A scan resolution of 72 dots per inch (dpi) and color depth of 48‐bit RGB were used.

Warm‐up effects arising from the warming up of the scanner lamp and subsequent heating of the scanner bed which, in turn, result in an initial decrease in optical density with repeated scanning, have been observed with some flatbed scanners operating in transmission mode.^(^ ^15^ ^,^ ^16^ ^)^ Five warm‐up scans were accordingly done prior to each batch of scans to stabilize the lamp and scanner bed temperature.

Previous investigations of EBT film have indicated that the measured optical density increases with repeated scanning of the same film.^(^ ^14^ ^)^ This effect is dependent on the initial level of irradiation, and has been attributed to the increase in temperature of the scan bed and film. To reduce or correct for the sensitivity of the measured dose to the properties of the film scanner, films were scanned with a minimal number of consecutive scans.

The manufacturer recommends that measurement be conducted with consideration to both red and blue channel data. However, Aland et al. and Kairn et al.^(^ ^7^ ^,^ ^11^ ^)^ recently suggested that the conversion of film optical density to measured dose should utilize red channel data only, without application of blue channel correction. In the present study, images were saved in TIFF format, and the red channel of the scan images was extracted and processed using ImageJ (NIH: National Institute of Health, Bethesda, MD). The row reading values were obtained as gray values, which corresponded to ADC values. Gray values over a 3× 3cm region of interest in the middle of the film pieces were averaged and evaluated.

Kairn et al.^(^ ^11^ ^,^ ^17^ ^)^ recommended that net optical density be measured on a pixel‐by‐pixel basis to account for local heterogeneities in EBT2 film. The present results are therefore presented in netOD, the calculation of which has been used in previous studies.^(^ ^15^ ^)^ For scanners that do not read optical density (OD) directly, netOD can be calculated as:

(1) $$\text{netOD}={\text{OD}}_{\text{exp}}-{\text{OD}}_{\text{unexp}}=\log_{10}(\frac{I_{unexp}-I_{bckg}}{I_{\exp}-I_{bckg}})$$

where $I_{unexp}$ and $I_{exp}$ are the readings for unexposed and exposed film pieces, and $I_{bckg}$ is the zero‐light intensity value obtained by measuring an opaque black sheet.

### D. Dose calibration curve

To obtain dose calibration curves for the five lots, 1 sheet from each lot was cut into 3× 3cm pieces, and 16 pieces of film were irradiated from 25 cGy to 400 cGy. Sixteen pieces of film from a similar area of the sheet were used to reduce the effect of local heterogeneity on the calibration curve. To reduce spatial variation in the measured dose, dose calibration curves were created by converting netOD values rather than gray values to the dose.^(^ ^13^ ^)^ Differences among dose calibration curves of the five lots were evaluated from 25 cGy to 400 cGy in 25 cGy increments.

### E. Intrasheet uniformity

To investigate intrasheet uniformity, 12 pieces of film from the same sheet were exposed to 2 Gy. Three sheets from each lot were used. Intrasheet uniformity was evaluated by the coefficient of variation of homogeneity for all pieces of a single sheet, and assessed in terms of gray value and netOD. Variations in irradiated film evaluated by gray value were compared to those in nonirradiated film to determine the correlation between irradiated and nonirradiated film.

### F. Intersheet uniformity

To evaluate intersheet uniformity, three sheets of the same lot number were irradiated at 2 Gy. Intersheet uniformity was evaluated by the coefficient of variation of homogeneity among the same piece numbers in the three sheets and assessed in terms of gray value and netOD.

The distribution of gray values and netOD obtained were analyzed. Intersheet uniformity was evaluated in terms of gray value and netOD.

### G. Dose evaluation

The netOD values of film pieces irradiated with 2 Gy were translated into dose values using the dose response curve. The obtained doses were compared to the applied dose of 2 Gy, and the differences in each lot number were evaluated.

### H. Dose dependence of heterogeneity

To investigate the dose dependence of heterogeneity, overall pieces in a single sheet of each lot were irradiated at 0.5 Gy, 2 Gy, and 3 Gy. The dose dependence of heterogeneity was evaluated by comparison of the intrasheet uniformity of nonirradiated films with that of irradiated films at 0.5 Gy, 2 Gy, and 3 Gy.

## III. RESULTS

### A. Dose calibration curve

**Figure 2:**
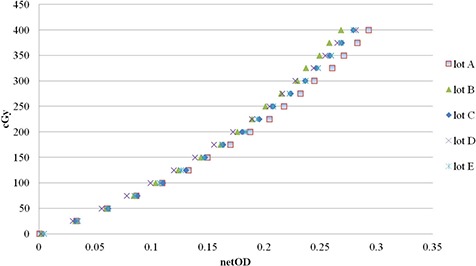
Dose‐response curve of the EBT2 film Lot Nos.F12170902A (lot A), A052810‐02A (lot B), A08161005A (lot C), A09171002 (lot D), and A05131001B (lot E). This is a sensitometric curve for conversion of netOD value to dose.

Dose calibration curves of five lots were created in the dose range of 25 cGy to 400 cGy with 25 cGy increment, as shown in Fig. 2. Differences between lots A and B, A and C, A and D, and A and E were 6.5%±2.7%, 3.1%±2.4%, 8.2%±1.9%, and 3.1%±2.6%, respectively; differences between lot B and C, B and D, and B and E were 3.3%±1.4%, 1.7%±4.0%, and 3.2%±1.4%, respectively; between lot C and D, and C and E, differences were 5.1%±3.9%, and 0.1%±2.2%, respectively; and between lot D and E they were 4.6%±3.6%.

### B. Intrasheet uniformity

**Figure 3:**
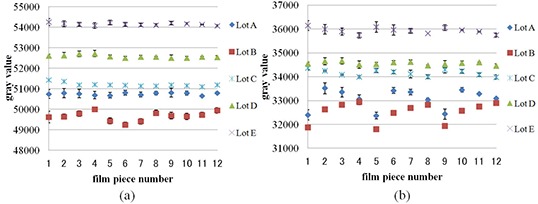
Gray value distribution of film pieces from lots A, B, C, D, and E before (a) and after (b) irradiation at 2 Gy.

Figure 3 shows the gray value variations before and after irradiation at 2 Gy. These data show average gray values of three sheets, and error bars indicate the coefficient of variation which represents intersheet uniformity. Regarding lots A and B, heterogeneity was increased by irradiation. Although intrasheet uniformity before irradiation was less than 0.4% in all lots, intrasheet uniformity after irradiation in lots A, B, C, D, and E was 1.5%, 1.3%, 0.4%, 0.2%, and 0.4%, respectively.

**Figure 4:**
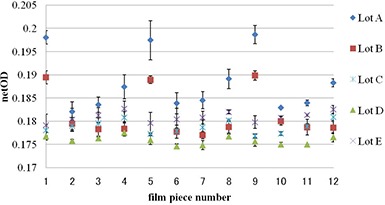
NetOD distribution of film pieces from lots A, B, C, D, and E after irradiation at 2 Gy.

Figure 4 shows netOD distribution. Intrasheet uniformity of lots A, B, C, D, and E was 3.0%, 2.7%, 0.8%, 0.5%, and 0.6% respectively. NetODs of lots A and B were high at areas 1, 5, and 9, whereas those of lots C, D, and E were low at areas 4, 8, and 12.

### C. Intersheet uniformity

Intersheet uniformity of all lots evaluated by gray value showed less than 0.6% before and after irradiation, and intersheet uniformity of all lots evaluated by netOD was less than 1.0% (Table 2). Intersheet uniformity evaluated by both gray value and netOD showed no significant difference.

**Table 2:** Intrasheet uniformity and intersheet uniformity obtained by netOD value.

	*Lot A*	*Lot B*	*Lot C*	*Lot D*	*Lot E*
Intrasheet Uniformity	3.0%	2.7%	0.8%	0.5%	0.6%
Intersheet Uniformity	1.0%	0.7%	0.4%	0.3%	0.8%

### D. Dose evaluation

**Figure 5:**
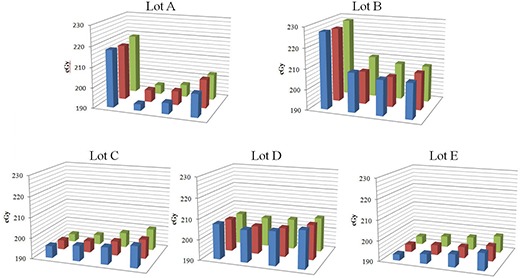
Dose distribution of lots A, B, C, D and E at 2 Gy. NetOD values of film pieces irradiated at 2 Gy were translated into dose values using the dose response curve. Film sheet position is displayed as in the schema in Fig. 1.

Figure 5 shows dose variations of film pieces irradiated at 2 Gy in lots A, B, C, D, and E. NetOD values were converted into the corresponding dose using the dose calibration curve shown in Fig. 2. The positions of bars in the graph correspond to the film sheet position in the Fig. 1 schema. Each lot showed a specific trend in dose distribution. Lots A and B showed the highest value at area 9. These lots had maximum differences of up to 7.8%, and 11.9% in comparison with 2 Gy. In contrast, lots C, D, and E showed the highest value at area 4. These lots had maximum differences of up to 3.1%, 3.6%, and 4.1% in comparison with 2 Gy. Intrasheet uniformity of lots A, B, C, D, and E were 4.8%, 4.3%, 1.1%, 0.7%, and 0.9%, respectively. In contrast, intrasheet uniformity evaluated by gray value was 4.6%, 5.5%, 1.4%, 0.6%, and 1.0%, respectively (data not shown). Intersheet uniformity of lots A, B, C, D, and E was 1.3%, 1.2%, 0.5%, 0.4%, and 1.2%, respectively (Table 3).

**Table 3:** Intrasheet uniformity and intersheet uniformity obtained by dose.

	*Lot A*	*Lot B*	*Lot C*	*Lot D*	*Lot E*
Intrasheet Uniformity	4.8%	4.3%	1.1%	0.7%	0.9%
Intersheet Uniformity	1.3%	1.2%	0.5%	0.4%	1.2%

### E. Dose dependence of heterogeneity

**Figure 6:**
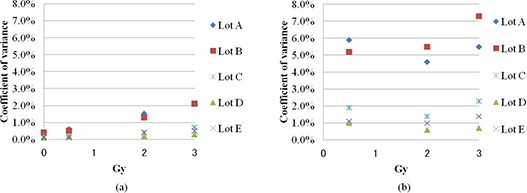
Intrasheet uniformity of lots A, B, C, D, and E after irradiation at 0.5, 2, and 3 Gy. Films were evaluated by gray value (a) and netOD (b).

Figure 6 shows the intrasheet uniformity of lots A, B, C, D, and E on irradiation at 0.5, 2, and 3 Gy. Lots were evaluated by both gray value and netOD. Dose dependence of film heterogeneity was observed in the gray value in all lots, but this was not observed in netOD in any lot. Intrasheet uniformity of lots A and B was greater than that of lots C, D, and E in both gray value and netOD evaluations.

## IV. DISCUSSION

In this study, we investigated the local heterogeneity of EBT2 films among various lots, as well as the dose dependency of heterogeneity in these lots. Results showed that the degree of heterogeneity in gray values differed in a dose‐dependent manner and varied among the lots.

Several studies have described local heterogeneity in EBT2 film. Kairn et al.^(^ ^17^ ^)^ reported that EBT2 suffered from some inconsistency in an early batch. Hartmann et al.^(^ ^9^ ^)^ reported significant heterogeneity, with intrasheet uniformity of 4.5% and a difference in dose determination due to heterogeneity of up to 6%. In the present study, two lots (A and B) showed significant local heterogeneity, and indicated large netOD values at the left side (areas 1, 5, and 9), which was compatible with the Hartmann study. The dose difference of lot B was biased toward the plus value (ranging from 0.5% to 11.9%) according to the film position of the calibration curve. Other film positions in calibration curve construction may lead to smaller differences in dose.

Richley et al.^(^ ^8^ ^)^ reported that intra‐ and intersheet uniformities were 1.2% and 0.6%, respectively. Three lots (C, D, and E) showed homogeneity, which was consistent with Aland et al.^(^ ^7^ ^)^ Their study reported that the degree of heterogeneity was smaller than that reported by Hartmann et al., noting intra‐ and intersheet uniformity of 0.7%, and 1.5%, respectively. Aland et al.^(^ ^7^ ^)^ also demonstrated that the effect of uniformity on gray value to dose conversion was up to 1.9%. To date, however, little data has appeared about homogeneity among several lots evaluated at the same time. We found that homogeneity tended to be associated with lot number.

In dose measurement, lots A and B showed higher values on left side of a film (areas 1, 5, and 9) than the other regions. In contrast, lots C, D, and E showed higher values on the right side. If the side with the highest value was excluded and the whole of the remaining side was used in dose measurement, intrasheet uniformity improved to 1.9%, 0.8%, 0.7%, 0.6%, and 0.5% in lots A, B, C, D, and E, respectively. Most importantly, maximum dose differences for lots A and B compared at 2 Gy significantly improved to 4.2% and 3.2%, respectively, which were similar to those for other lots. These results suggest that the use the middle region of the film improves dose verification accuracy with EBT2 film.

Although lots B and C had the same expiration date, they differed in their degree of intrasheet uniformity. This finding suggests that film homogeneity was independent of expiration date, but dependent on lot number, and that film homogeneity continues to be variable, despite improvements in lot uniformity overtime.

The manufacturer recommends that EBT2 film scans be corrected using red and blue channels. However, Kairn et al.^(^ ^11^ ^)^ reported that blue channel correction increased noise in the resulting dose images, leading to an increase in dose uncertainty of ±7.5% or ±1.2% with or without blue channel correction, respectively. These authors recommended that the films be analyzed using netOD without blue channel correction to ensure the production of dose images with minimal film heterogeneity effects. In the present study, dose variation was greater by gray value than by netOD analysis in lot B, but not significantly different in other lots. On the other hand, intrasheet uniformity evaluated by gray value indicated dose dependence, whereas that by netOD value indicated dose independence. These results suggested that evaluation by netOD was the appropriate analysis method for almost all lots of EBT2.

## V. CONCLUSIONS

EBT2 should be examined for uniformity by sheet position in each lot number before clinical use. Close attention to the sheet location which shows the least uniformity will likely provide sufficient film homogeneity for clinical use independent of lot.

## ACKNOWLEDGMENTS

This study was supported by Grants‐in‐Aid for Scientific Research (22791194) from the Japan Society for the Promotion of Science.

## References

[1] Fuss M , Sturtewagen E , De Wagter C , Georg D . Dosimetoric characterization of GafChromic EBT film and its implication on film dosimetry quality assurance. Phys Med Biol. 2007;52(14):4211–25. 17664604 10.1088/0031-9155/52/14/013

[2] Anjum MN , Parker W , Ruo R , Afzal M . Evaluation criteria for film based intensity modulated radiation therapy quality assurance. Phys Med. 2010;26(1):38–43. 19620015 10.1016/j.ejmp.2009.06.002

[3] Zeidan OA , Stephenson SAL , Meeks SL , et al. Characterization and use of EBT radiochromic film for IMRT dose verification. Med Phys. 2006;33(11):4062–72. 10.1118/1.2360012 17153386

[4] Todorovic M , Fischer M , Cremers F , Thom E , Schmidt R . Evaluation of GafChromic EBT prototype B for external beam dose verification. Med Phys. 2006;33(5):1321–28. 16752567 10.1118/1.2188077

[5] Arjomandy B , Tailor R , Anand A , et al. Energy dependence and dose response of Gafchromic EBT2 film over a wide range of photon, electron, and proton beam energies. Med Phys. 2010;37(5):1942–47. 20527528 10.1118/1.3373523

[6] Sutherland JG and Rogers DW . Monte Carlo calculated absorbed‐dose energy dependence of EBT and EBT2 film. Med Phys. 2010;37(3):1110–16. 20384246 10.1118/1.3301574 PMC2837726

[7] Aland T , Kairn T , Kenny J . Evaluation of a Gafchromic EBT2 film dosimetry system for radiotherapy quality assurance. Australas Phys Eng Sci Med. 2011;34(2):251–60. 21465275 10.1007/s13246-011-0072-6

[8] Richley L , John AC , Coomber H , Fletcher S . Evaluation and optimization of the new EBT2 radiochromic film dosimetry system for patient dose verification in radiotherapy. Phys Med Biol. 2010;55(9):2601–17. 20393235 10.1088/0031-9155/55/9/012

[9] Hartmann B , Martisiková M , Jäkel O . Homogeneity of Gafchromic EBT2 film. Med Phys. 2010;37(4):1753–56. 20443496 10.1118/1.3368601

[10] Ezzell GA , Burmeister JW , Dogan N , et al. IMRT commissioning: multiple institution planning and dosimetry comparison, a report from AAPM Task Group 119. Med Phys. 2009;36(11):5359–73. 19994544 10.1118/1.3238104

[11] Kairn T , Aland T , Kenny J . Local heterogeneities in early batches of EBT2 film: a suggested solution. Phys Med Biol. 2010;55(15):L37–L42. 20616403 10.1088/0031-9155/55/15/L02

[12] Martisíková M , Ackermann B , Jäkel O . Analysis of uncertainties in GafchromicEBT film dosimetry of photon beams. Phys Med Biol. 2008;53(24):7013–27. 19015581 10.1088/0031-9155/53/24/001

[13] Saur S and Frengen J . GafChromic EBT film dosimetry with flatbed CCD scanner: a novel background correction method and full dose uncertainty analysis. Med Phys. 2008;35(7):3094–101. 10.1118/1.2938522 18697534

[14] Lynch BD , Kozelka J , Ranade MK , Li JG , Simon WE , Dempsey JF . Important considerations for radiochromic film dosimetry with flatbed CCD scanners and EBT GAFCHROMIC film. Med Phys. 2006;33(12):4551–56. 17278806 10.1118/1.2370505

[15] Devic S , Seuntjens J , Sham E , et al. Precise radiochromic film dosimetry using a flatbed document scanner. Med Phys. 2005;32(7):2245–53. 10.1118/1.1929253 16121579

[16] Paelinck L , De Neve W , De Wagter C . Precautions and strategies in using a commercial flatbed scanner for radiochromic film dosimetry. Phys Med Biol. 2007;52(1):231–42. 17183138 10.1088/0031-9155/52/1/015

[17] Kairn T , Kenny J , Crowe SB , et al. Modeling a complex micro‐multileaf collimator using the standard BEAMnrc distribution. Med Phys. 2010;37(4):1761–67. 20443498 10.1118/1.3355873

